# Defining benchmarks for tolerable risk thresholds in cancer screening: Impact of HPV vaccination on the future of cervical cancer screening

**DOI:** 10.1002/ijc.33178

**Published:** 2020-07-16

**Authors:** Joseph E. Tota, Sandra D. Isidean, Eduardo L. Franco

**Affiliations:** ^1^ Division of Cancer Epidemiology and Genetics National Cancer Institute Rockville Maryland USA; ^2^ Department of Oncology McGill University Montreal Quebec Canada; ^3^ Department of Pharmacoepidemiology Merck Research Laboratories West Point Pennsylvania USA

**Keywords:** cervical cancer, HPV vaccination, screening, tolerable risk

## Abstract

The performance of cervical cancer screening will decline as a function of lower disease prevalence—a consequence of successful human papillomavirus (HPV) vaccination. Replacement of cytology with molecular HPV testing as the primary screening test and adoption of risk‐based screening, with less intense screening of vaccinated individuals and initiated at older ages is expected to improve efficiency. However, policy officials may decide to further reduce or eliminate screening as the ratio of benefits to harms continues to decline. To evaluate the level of risk currently tolerated for different cancers in the United States (ie, for which clinical guidelines do not recommend secondary prevention though effective screening methods exist), we used US cancer registry data to compare incidence (2008‐2012) and survival (1988‐2011) associated with different cancers for which organized screening is recommended and not recommended. The most common cancer at ages 70 to 74 years (ie, age group with highest cancer incidence and reasonable life expectancy to consider screening in the US) satisfying Wilson and Jungner's classic screening criteria was vulvar cancer (incidence = 9/100 000 females). In comparison, the incidence of cervical cancer among females 65 years of age (the upper recommended age limit for screening) was 13 cases per 100 000 females (low as a reflection of effective screening), whereas 10‐year survival was 66% (similar to vulvar cancer at 67%). Our approach of defining tolerable risk in cancer screening could help guide future decisions to modify cervical screening programs.

**Abbreviations**
ACS: American cancer society
CIN: cervical Intraepithelial neoplasia
HPV: human papillomavirus
QALY: quality‐adjusted life‐year
SEER: surveillance, epidemiology, and end results
USD: US dollars
USPSTF: United States preventive services task force

## 1 INTRODUCTION

In 2006, *Gardasil* (MSD) was the first prophylactic human papillomavirus (HPV) vaccine approved in the United States and shortly after, in 2009, *Cervarix* (GlaxoSmithKline) was also approved. Both vaccines target two of the 12 oncogenic HPV types (HPVs 16 and 18), responsible for approximately 60% to 70% of cervical cancer cases worldwide.^1^ Prior to marketing these vaccines, investigators had already begun to consider the impact that vaccination would have on cervical cancer screening.^2^ Modeling studies revealed that as lesion prevalence declined, the positive predictive value of cytology (ie, probability that patients with a positive screening test truly have cervical precancer/cancer) would become too low to maintain it as the primary screening test.^2^ As the first vaccinated cohorts now become eligible for screening, there is urgency to introduce an alternative approach. Most experts agree that cytology should be replaced with more sensitive HPV DNA testing, perhaps reserving cytology (a test with excellent specificity) for triaging HPV‐positive women for referral to colposcopy.^3^, ^4^, ^5^

The recent arrival of *Gardasil 9* (MSD), which targets five other oncogenic HPV types, responsible for an additional ~20% of cervical cancer cases globally, will have an even greater impact on disease prevalence and screening performance.^1^, ^6^ Nontargeted HPV types now account for <10% of cervical cancer cases globally,^1^ and as the risk of cervical cancer continues to decline in vaccinated populations, policy officials may consider reducing or eliminating cervical cancer screening.

It remains unclear at what point (risk level) cervical screening may be reduced or discontinued in vaccinated women. To identify benchmarks for tolerable risk that currently exist, we selected cancers that may be good candidates for screening according to established criteria,^7^ and compared burden (incidence and survival) in relation to current recommendations. The cancer with the highest incidence that we do not screen for (though effective screening methods exist) might then be used for defining this threshold. Data for these comparisons were obtained from the US Surveillance, Epidemiology and End Results (SEER) Program.

## 2 CRITERIA AND SELECTED CANCERS FOR COMPARISON

In deciding whether to screen for a disease, there are important factors to consider. In 1968, Wilson and Jungner proposed the following 10 criteria to judge the suitability of a screening program: (a) disease is an important health problem, (b) natural history is adequately understood, (c) latent/early symptomatic stage exists, (d) suitable test/examination is available, (e) test/exam under consideration is acceptable to the population, (f) accepted treatment exists, (g) facilities for diagnosis and treatment are available, (h) agreed policy on whom to treat, (i) cost of screening outweighed by benefits and (j) case‐finding is a continuous process.^7^ In Table 1, we present our subjective evaluation of whether selected cancers satisfy each of these criteria, ranging from −/+ (cancer type either equivocally or does not satisfy criteria) to ++ (cancer type satisfies criteria).

**TABLE 1:** Criteria for screening for various cancers (based on Wilson and Jungner's classic screening criteria)

	Cervix	Vulva	Vagina	Ovary	Breast		Oral (mouth)^a^	Anal^a^	Colorectal^a^	Melanoma		Lung^a^	Prostate	Thyroid^a^
					Men	Women				Men	Women			
Incidence^b^	++^c^	++	+	++	+	+++	++	+	+++	+++	++	+++	+++^c^	++
Survival^b^	++	++	+++	+++	++	+	+++	++	++	+	+	+++	+	+
Natural history of condition adequately understood	++	++	+	+	+/−	++	++	++	++	++	++	+	++	+/−
Recognizable latent or early symptomatic stage exists	++	++	++	+	+/−	++	++	++	++	+	+	++	++	+/−
Suitable test or examination available^d^	++	++	++	+/−	++	++	++	++	++	++	++	++	++	++
Test acceptable to population^d^	++	++	++	++	+	++	++	++	++	++	++	++	++	++
Accepted treatment for disease exists	Yes (conditional on availability of secondary and tertiary care facilities)													
Facilities for diagnosis and treatment available^e^														
Agreed policy on whom to treat as patients	++	++	++	+	+	++	++	++	++	++	++	++	+	+
Potential harms of undergoing screening^f^	Yes													
Cost‐effectiveness^g^	++	+/−	+/−	+/−	+/−	++	+/−	+	++	++	++	+	+/−	+/−
Case‐finding a continuing process	Yes (conditional on risk group or antecedents)													

^a^ Estimates for men and women were collapsed because of comparability in incidence and survival statistics.

^b^ Incidence and survival data were used to reflect Wilson and Jungner's first criterion (ie, whether the disease at hand is an important health problem). For incidence: +: <10, ++: >10, <100, +++: >100 per 100 000. For survival: +: >75%, ++: <75%, >50%, +++: <50%.

^c^ Incidence directly affected by screening (downwards for cervix and upwards for prostate).

^d^ Includes only tests that have been in widespread use and/or have clinical application in specific circumstances. Diagnostic work‐up procedures not considered.

^e^ Includes management decisions and diagnostic work‐up.

^f^ Considers psychological harms and potential morbidity and risks during the entire screening process, including diagnostic work‐up and treatment.

^g^ Cancers for which organized, guideline‐driven screening programs currently exist were considered highly cost‐effective (ie, ++). Cancers for which screening guidelines exist for high‐risk subgroups only, or in opportunistic settings were considered moderately cost‐effective (ie, +). Uncertainty regarding the cost‐effectiveness of screening was considered equivocally cost‐effective (ie, +/−).

In addition to cervical cancer,^8^, ^9^ the American Cancer Society (ACS) and the United States Preventive Services Task Force (USPSTF) recommend screening for breast,^10^, ^11^ colorectal^12^, ^13^ and lung cancers.^14^, ^15^ The USPSTF recommends that only older current and former smokers (having quit within the past 15 years), with at least 30 pack‐years of smoking, undergo screening for lung cancer.^15^ For cervical, breast and colorectal cancers, age and gender are often the only factors considered in selecting patients for screening.

For most other cancers that we consider, the tissue at risk is easily accessible and amenable to early detection but systematic screening is not recommended (eg, skin, thyroid, oral, vulvar, vaginal and anal cancers). We also include prostate and ovarian cancers for comparison; both of which are common but have different survival outcomes. The data that we used for this analysis are available from the SEER Research Database: https://seer.cancer.gov/.

## 3 ANNUAL CANCER INCIDENCE (2008‐2012) AND SURVIVAL (1988‐2011) IN THE UNITED STATES

### 3.1 Organized screening recommended: Cervix, colon, breast and lung cancers

For cervical and colorectal cancers, detection and treatment of precancerous lesions or polyps are possible before they become invasive. The incidence of these cancers is therefore expected to be higher in the absence of screening. On the other hand, early detection is the goal of screening for breast and lung cancers and the incidence of these cancers may be lower in the absence of screening due to overdiagnosis.^16^, ^17^, ^18^

**FIGURE 1:**
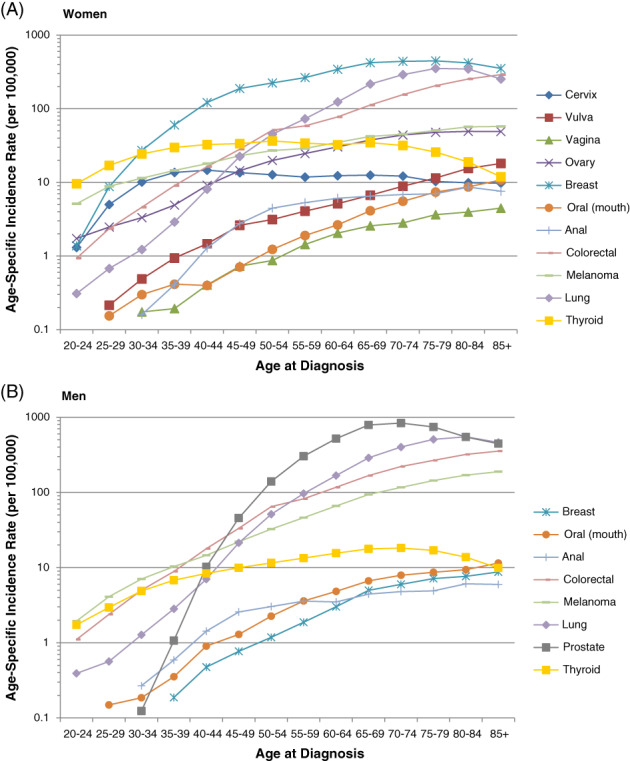
Age‐specific incidence rates of various cancer sites, A, in women and B, in men according to United States SEER registry data (2008‐2012)

In the United States, it is recommended that females aged 21‐65 years regularly undergo screening for cervical cancer at least once every 3 years via cytology, or every 5 years (if over Age 30) via co‐testing with cytology and HPV DNA testing (recommended by both ACS and USPSTF) or HPV testing alone (recommended by USPSTF only).^8^, ^9^ Based on US SEER18 registry data (years 2008‐2012), the average annual incidence of cervical cancer among females eligible for screening in the United States ranged from 1 (Age 21) to 13 cases (Age 65) per 100 000 females (Figure 1).

For colorectal cancer, screening is strongly recommended for individuals aged 50 to 75 years,^12^, ^13^ and average annual incidence at these ages ranged from 51 to 162 cases (females) and 64 to 233 cases (males) per 100 000 individuals, respectively. Due to the recent rising incidence of colorectal cancer in younger individuals in the United States,^19^ the ACS issued a qualified recommendation in 2018 to initiate screening at Age 45.^13^ The recommendation was issued as “qualified” rather than “strong” because evidence regarding the balance of benefits and harms remains lacking.^13^

Focusing on breast cancer, guidelines are consistent in recommending that screening end at Age 75 but differ on start age. The ACS recommends it begin at Age 40,^10^ whereas the USPSTF recommends Age 50.^11^ Average annual incidence of breast cancer at ages 40 and 75 years ranged from 122 to 434 cases per 100 000 females, respectively. Finally, lung cancer screening is recommended for individuals aged 55 to 80 years^14^, ^15^ and average annual incidence at these ages ranged from 73 to 354 cases (females) and 99 to 520 cases (males) per 100 000 individuals, respectively.

**FIGURE 2:**
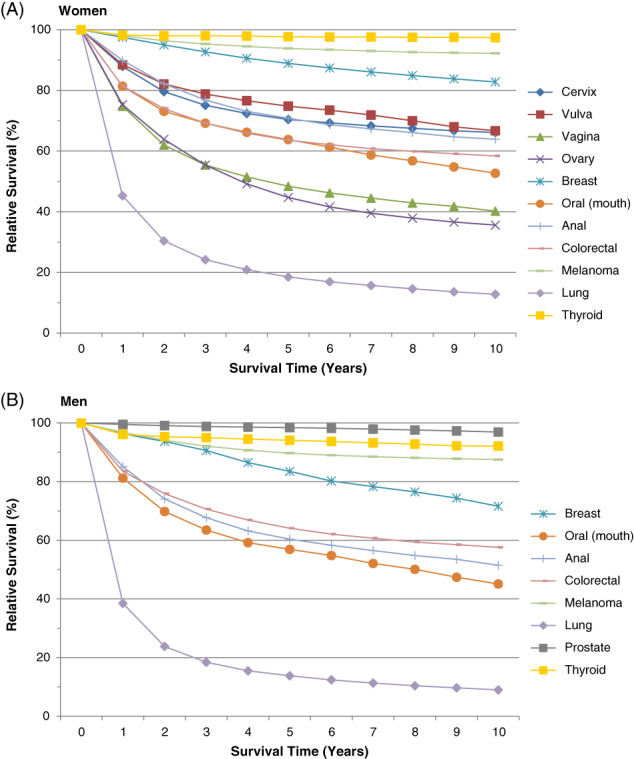
Relative survival by survival time (years) for various cancer sites, A, in women and B, in men according to United States SEER registry data (1988‐2011)

Among females diagnosed with cervical cancer, overall 10‐year survival was 66% (based on US SEER registry information for the years 1988‐2011; Figure 2). Survival was low for lung cancer (13% among females; 9% among males), high for breast cancer (83% among females) and moderate for colon cancer (58% in both genders).

### 3.2 Organized screening not recommended: Prostate, thyroid, ovarian, skin, oral, vulvar, vaginal and anal cancers

We also present incidence and survival for selected cancers for which organized screening is not recommended (Figures 1 and 2, respectively). In our assessment of these cancers (Table 1), we determined that while the strength of evidence varies, screening *may* be possible for all cancers except ovarian, due to the lack of a suitable screening test. In examining possible benchmarks for tolerable risk, we report the highest cancer incidence observed across all ages. However, given that risk generally increases with age and it is unlikely that screening would be recommended for individuals with low remaining life expectancy, we also present incidence for individuals aged 70 to 74 years (values below, in parentheses).

Among females, the highest observed cancer incidence (cases per 100 000 individuals) was 56 (44) for melanoma, 51 (45) for ovarian, 18 (18) for thyroid, 18 (9) for vulvar, 10 (5) for oral, 8 (6) for anal and 4 (3) for vaginal. The 10‐year survival for these cancers was 92%, 35%, 97%, 67%, 52%, 63% and 41%, respectively. Similarly, among males, the highest observed cancer incidence was 895 (895) for prostate, 182 (113) for melanoma, 37 (32) for thyroid, 12 (8) for oral, 9 (6) for breast and 6 (5) for anal. The 10‐year survival for these cancers was 97%, 87%, 92%, 45%, 72% and 52%, respectively.

## 4 COMPARISON OF CANCER RISK AND SURVIVAL

Among cancers for which organized screening is currently recommended, cervix is by far the least common (Figure 1). Overall survival is also high compared to the other cancers that we examined. Among females, vulvar, breast, thyroid and melanoma cancers have better prognoses (Figure 2).

In our initial attempt to identify cancers that could serve as benchmarks for tolerable risk, we focused on those with high incidence (≥10 cases per 100 000). According to this criterion, we identified vulvar, thyroid, melanoma, prostate and ovarian cancers. Although ovarian cancer has poor prognosis, little is known about its natural history, there is no recognizable latent/early symptomatic stage, and no suitable screening test exists (Table 1). Thyroid, melanoma and prostate cancers, despite being common, have good prognoses (>85%). For prostate cancer, the natural history is reasonably understood (but not to the extent of cervical, vulvar and vaginal cancers), there is no recognizable latent/early symptomatic stage, and no agreed policy on whom to treat.

In addition to incidence, the definition of benchmarks of tolerable risk must also consider clinical outcomes, which implies focusing on cancers with poor overall prognoses (10‐year survival <80%). Excluding ovarian cancer, the cancers with the highest incidence at ages 70 to 74 years for which we currently do not screen were identified to be vulvar (9 cases per 100 000 females) and oral cancers (6 and 8 cases per 100 000 individuals in females and males, respectively). While 10‐year survival for cervical cancer (66%) was substantially higher compared to survival for oral cancer (52% among females and 45% among males), survival for cervical cancer was similar compared to survival for vulvar cancer (67%).

## 5 IMPLICATIONS FOR SCREENING POLICY

In this analysis comparing cervical cancer burden with other cancers for which organized screening programs exist (breast, colorectal and lung) and do not exist (ovarian, prostate, thyroid, skin, oral, vulvar, vaginal and anal), cervical cancer ranked poorly in the former group (ie, lowest incidence and second best survival) and only moderately in the latter group (ie, among the lowest incidence and generally similar survival)—a reflection of effective screening that has prevailed in the US for several decades. Among cancers that we do not currently screen for but satisfy Wilson and Jungner's criteria (Table 1), vulvar cancer has the highest incidence (9 cases per 100 000 at ages 70‐74 years) and is arguably the best candidate for defining tolerable risk. Vulvar squamous cell carcinoma has a comparable precancerous lesion stage to cervical cancer that is amenable to detection via exfoliative cytological screening or molecular HPV testing of exfoliated cells and diagnostic biopsies can be obtained during a pelvic examination. During an exam, cytology may be performed to identify morphologic features of vulvar lesions and has the advantage of being guided by visual examination with smears taken from all abnormal‐looking areas. As a result, performance could be even better compared to cervical screening using cytology.^20^ While a higher proportion of vulvar lesions may be attributed to HPV among younger patients,^21^ we do not expect that performance of cytology screening would differ according to HPV status.

Despite similar incidence and survival, relative to cervical cancer, there are no public health guidelines compelling healthcare providers to screen for vulvar cancer. These conditions make this disease a suitable benchmark for tolerable risk, which could be applied to decisions on when to stop cervical cancer screening in the post‐HPV vaccination era. In settings with effective cervical screening in place, we may estimate potential cervical cancer risk if screening were to be discontinued using incidence information for high‐grade cervical precancerous lesions (identified/treated via screening) and applying progression rates taking into account variability according to type‐specific HPV status, for example, higher rate of progression for HPV16‐positive lesions compared to lesions caused by other HPV types.^22^ While no recent study has evaluated risk of progression from cervical intraepithelial neoplasia grade 3 (CIN3) to invasive cancer, an unethical clinical study conducted from 1965 to 1974 in New Zealand found that approximately one‐third of CIN3 lesions progressed to cancer when treatment was withheld.^23^

In addition to providing excellent protection against cervical cancer, we also expect a reduction in the incidence of vulvar cancer due to vaccination. Approximately 25% of vulvar cancer cases globally are attributable to HPV^24^ and among these cases, approximately 90% are caused by the seven oncogenic HPV types targeted by Gardasil 9.^25^ In settings with successful cervical cancer screening programs, approximately 60% to 80% of cervical cancer cases may be prevented.^26^, ^27^ If screening for vulvar cancer were to be recommended and successfully implemented with identification and treatment of vulvar intraepithelial neoplasia lesions, then we may expect a decline in the incidence of vulvar cancer that is similar. While we should acknowledge that reductions in vulvar cancer incidence may be achieved through vaccination and potentially screening, this does not impact our assessment and conclusions regarding current tolerable risk thresholds.

Within a few decades from now, owing to the success of vaccination enabling protection of most birth cohorts, the annual incidence of cervical cancer will likely drop below current incidence levels for vulvar cancer, across all age groups. Arguably, screening could then be eliminated or conducted very infrequently to reduce costs and associated harms.

Preterm delivery and low birth weight are important obstetric outcomes associated with excisional treatment of cervical precancerous lesions.^28^, ^29^ Other potential harms that may result from biopsy and treatment include anxiety, pain, bleeding and discharge.^30^ A recent study evaluating cervical screening benefits (incidence/mortality) and related harms suggests a substantial reduction in screening‐related harms may be achieved in the United States by adopting a less intense screening approach that is similar to recommendations in the Netherlands (ie, cytology screening every 5 years), without any loss in benefit.^30^ Investigators reported that the number of preterm deliveries attributable to screening could have been reduced from 5300 to 2100 and that other harmful events (ranging from anxiety to severe bleeding) could have been reduced from 8 663 000 to 3 202 000 among the 90 905 000 women aged 21 to 65 eligible for screening in the United States in 2007.^30^

Recent microsimulation studies evaluating optimal cervical screening approaches for vaccinated individuals in the United States^31^ and England^32^ indicate that screening may safely be initiated at older ages (eg, 35 years) with extended intervals using HPV testing (eg, ≥10 years).^31^, ^32^ However, these models do not consider the negative effect of vaccination on the positive predictive value of screening tests, which could fall below 2%, as the prevalence of high‐grade precancerous lesions markedly declines in vaccinated populations.^33^ Among females aged 21 to 24 years undergoing screening at Kaiser Permanente Northern California, 3‐year risk of cervical intraepithelial neoplasia grade 3 or greater following high‐grade cytology results was reported to be much lower among vaccinated individuals (received before Age 18) compared to unvaccinated individuals (3.70% vs 0.99%, respectively).^34^ These results suggest that we should consider raising the age of screening initiation among vaccinated females.

In 2008, the concept of developing a risk‐based strategy to manage patients in cervical cancer screening was developed by Castle et al,^35^ and refined by Katki et al.^36^ The approach is appropriately referred to as “equal management of equal risks” and advocates applying similar management based on different combinations of test results conferring the same cancer risk, a concept that is now incorporated in new professional guidelines.^37^ Although this concept of “benchmarking” risk was intended to be specific to cervical cancer, it relates to our concept of establishing tolerable risk thresholds in cancer screening. However, one of the issues in trying to identify a tolerable risk threshold is that it does not consider costs—a key criterion proposed by Wilson and Jungner.^7^

In the same way that we consider an acceptable benchmark for risk of cervical cancer, we may also determine the benchmark for deciding if an intervention is cost‐effective. For example, is it $50 000, $100 000 or $150 000 US dollars (USD) per quality‐adjusted life‐year (QALY) gained? Outside of the context of an explicit resource constraint, this is a difficult question to address; however, Neumann and colleagues suggest using either $100 000 or $150 000 USD.^38^ If we are able to compare costs per QALY for different interventions, then substituting less cost‐effective for more cost‐effective ones would become more objective and likely more common, especially as resources become increasingly constrained.^39^

## 6 CONCLUSIONS AND PERSPECTIVE

In the context of cervical cancer prevention, there are two important questions: (a) “How can we achieve higher vaccination coverage?” and (b) “Once higher coverage is attained, should cervical cancer screening be discontinued?” The first question is outside the scope of this analysis, but our rationale is related to the second question. If policy decisions should eventually discontinue or reduce cervical cancer screening based on an unfavorable cost‐benefit ratio in a future that includes high vaccination coverage, then it stands to reason that today's policymaking should allocate more resources towards reaching this goal. But the most likely scenario is that screening intensity will vary across settings as a reflection of different nations’ budgets for health care expenditures and societal variations in risk tolerance.

The process of creating screening recommendations has historically focused on evaluating evidence related to each cancer site individually. To the best of our knowledge, guideline committees do not compare risk across cancer sites. In 2015, Whitham and Kulasingam evaluated risk of cervical cancer at and after the recommended age to begin and end screening in relation to risk of breast and colorectal cancers, revealing the higher propensity to screen for cervical cancer despite lower risk.^40^ Our analysis comparing incidence and survival across cancer sites for which screening is recommended and not recommended, to assess tolerable risk, is an extension of this approach.

At the time this article was under consideration, the preventive measures taken in response to the COVID‐19 epidemic had begun to adversely affect the entire range of activities related to cancer control, prevention and care. Justifiably, controlling the epidemic is a much greater priority, which must receive the necessary personnel and material resources to ensure a successful operation. Public health activities related to cancer screening and prevention, typically carried out in outpatient clinics, were considered of lower priority and were thus scaled back to conserve resources, as well as to prevent exposure of cancer screening participants to SARS‐CoV‐2. In addition, adoption of policy decisions that could improve the uptake and quality of cancer screening services may be delayed because of the epidemic. Likewise, coverage of HPV vaccination is likely to suffer in consequence of behavioral changes related to health promotion. Although a disruption in cancer control activities is likely to lead to an increase in incidence of cancers preventable via screening and vaccination it does not invalidate the arguments we presented in this article.

## CONFLICT OF INTEREST

J. E. T. is an employee of Merck Sharp & Dohme Corp., a subsidiary of Merck & Co., Inc., Kenilworth, NJ, USA but completed all work associated with this manuscript while employed at the National Cancer Institute. E. L. F. does not have a conflict of interest with the content of this manuscript but has occasionally served as a consultant to Merck and GSK on HPV vaccines, and to Roche and BD on HPV diagnostics. His institution received unrestricted grants from Merck and Roche. S. D. I. declared no potential conflicts of interest.
